# Effects of Combined MSWI Flue Gas Treatment Residues, Fly Ash and Boiler Dust on the Properties of Cement Mortars

**DOI:** 10.3390/ma19204393

**Published:** 2026-10-19

**Authors:** Malgorzata Ulewicz, Alina Pietrzak

**Affiliations:** Faculty of Civil Engineering, Czestochowa University of Technology, Dabrowskiego 69 Street, PL 42-201 Czestochowa, Poland; alina.pietrzak@pcz.pl

**Keywords:** cement mortar, MSWI residues, boiler dust, fly ash, mechanical strength, freeze–thaw resistance

## Abstract

This study evaluated the feasibility of using two hazardous residue streams from municipal solid waste incineration (MSWI) as partial substitutes for conventional constituents of cement mortars. A mixture of solid residues from flue gas treatment and fly ash (MSWI-SW/FA; 19 01 07* and 19 01 13*) was used as a partial replacement for natural sand, whereas boiler dust containing hazardous substances (MSWI-BD; 19 01 15*) was used as a partial cement replacement. The residues were incorporated separately and simultaneously at different replacement levels. Mortar consistency, compressive and flexural strength after 7 and 28 days, water absorption, freeze–thaw resistance, and microstructure were evaluated. At low replacement levels, mechanical properties remained close to those of the reference mortar. After 28 days, the best mixture containing both residues reached compressive and flexural strengths of 50.68 and 9.01 MPa, compared with 52.23 and 9.36 MPa for the reference mortar. Increasing MSWI-BD content reduced strength and increased water absorption, whereas MSWI-SW/FA improved freeze–thaw resistance. The most favorable freeze–thaw performance was obtained for MIX 2.93/17.07, with only a 3.20% reduction in compressive strength after 25 cycles. Mortar performance depended strongly on residue type, replacement level, and relative proportion.

## 1. Introduction

Efficient use of raw materials and the management of waste streams are important components of sustainable production systems [1]. In the construction materials sector, one approach to implementing this concept is the use of waste materials as secondary raw materials to partially replace conventional constituents in cement-based composites [2]. In this context, residues generated during the thermal treatment of municipal solid waste constitute a particularly important group, as they contain mineral components with potential material value, while at the same time they may contain hazardous substances that limit their direct utilization.

The municipal solid waste incineration (MSWI) process, when combined with energy recovery, reduces the amount of waste directed to landfills while enabling the recovery of heat generated during combustion and its conversion into useful thermal and electrical energy through a combined heat and power system. At the same time, the process generates mineral residues that require further management. Solid MSWI residues consist primarily of bottom ash, accounting for approximately 25–30% of the mass of the incinerated waste, as well as considerably smaller quantities of fly ash and flue gas treatment residues, corresponding to approximately 2.5–3.0% of the waste input mass [3]. The individual residue streams differ in terms of their point of generation within the installation, chemical and mineralogical composition, particle size distribution, and contents of soluble salts and metals, which directly affect their potential for further utilization. Their properties are also strongly dependent on their source, and significant differences in chemical and mineralogical composition may occur not only between different MSWI plants but also between successive batches of material originating from the same installation [4]. Fine residues generated in the boiler section and in dust removal and flue gas cleaning systems represent a particular challenge. According to the European waste classification [5], the corresponding hazardous waste streams include, among others, solid wastes from gas treatment (19 01 07*), fly ash containing dangerous substances (19 01 13*), and boiler dust containing dangerous substances (19 01 15*). Compared with bottom ash and other coarser mineral fractions, these materials may contain higher concentrations of soluble chlorides and sulfates, as well as elevated levels of certain metals, which limits their direct material utilization [6,7,8]. Therefore, assessment of their utilization potential should consider both their possible function as secondary mineral raw materials and the possibility of reducing the mobility of hazardous constituents after incorporation into the resulting material.

One possible solution is to incorporate MSWI residues into a cementitious matrix, which enables their utilization as a composite constituent while potentially reducing the mobility of the hazardous components they contain. The effectiveness of this approach has been demonstrated for MSWI ashes and other residues, with substantial reductions in the leaching of chlorides, sulfates, and selected heavy metals following their immobilization in cement-based composites [9,10]. These findings justify further research into the use of MSWI residues in cementitious materials; however, the technical properties of the resulting composite and its behavior under service conditions must be evaluated separately for each specific type and composition of waste.

From the perspective of the circular economy, the use of waste materials in cement-based composites can simultaneously reduce the consumption of primary raw materials and the amount of material directed to landfill. Such utilization has been demonstrated for a wide range of waste streams, including ceramic waste [11,12], biomass combustion fly ash [13,14], metallurgical industry waste such as slags and sludges [15,16], and waste glass used as a partial replacement for natural aggregate in cement mortars [17]. Composites containing two different waste materials simultaneously have also been investigated, as exemplified by the use of polyamide waste in combination with biomass combustion fly ash [18]. From a materials engineering perspective, therefore, the key issue is not merely the incorporation of waste into a composite, but rather the determination of the relationships between its composition, content, and function in the mixture and the resulting microstructure, mechanical properties, and durability of the material.

In the case of municipal solid waste incineration residues, most previous studies have focused on the use of municipal solid waste incineration bottom ash (MSWI-BA) and slag. These materials have been used as partial replacements for natural aggregate in concrete [19,20,21] and as partial replacements for cement [22,23]. Pre-treated MSWI bottom ash, subjected to thermal and chemical treatment, has also been used as a partial replacement for natural sand in cement mortars [24]. In more recent research, Vilarinho et al. [25] investigated MSWI bottom ash as an aggregate replacement, a partial binder replacement, and a filler in cement mortars, confirming a strong dependence of composite properties on the mode of waste utilization and the replacement level. Beyond conventional Portland-cement-based systems, MSWI residues have also been investigated in alternative cementitious binders. For example, Pavlík et al. [26] incorporated MSWI bottom ash into magnesium potassium phosphate cement composites and demonstrated its potential for modifying technical properties while also addressing heavy metal immobilization. The use of MSWI bottom ash in road construction has also been investigated, indicating its potential application in road subbase layers, provided that appropriate technical properties and a limited content of soluble salts are ensured [27]. However, the effect of incorporating these materials into composites strongly depends on their particle size distribution, pre-treatment method, mineralogical composition, presence of metallic aluminum, and replacement level [3]. The source of the material is also an important factor, as comparisons of slags originating from different Polish installations have shown clear differences in their physicochemical properties [28]. The importance of waste composition is also evident in relation to composite durability. Thomas and Slosarczyk [29], in their study of mortars containing MSWI slag, demonstrated the influence of this material on mechanical properties, water absorption, and resistance to cyclic freezing and thawing, while also indicating the potential significance of chlorides and sulfates for microstructural changes under cyclic exposure.

A second extensively investigated group comprises MSWI fly ashes (MSWI-FA). In cementitious materials, they have been studied as partial cement replacements in pastes and mortars [30,31,32], as well as substitutes for cement or natural sand [33]. In a broader approach to MSWI residue valorization, Zhang et al. [34] produced an eco-admixture from a thermally treated mixture of bottom ash, boiler ash, and air pollution control lime, which was subsequently used as a partial cement replacement in concrete. Another research direction involves the use of MSWI-FA as a raw material in cement production [35,36], as well as a component or precursor in alkali-activated materials [8,37]. However, the utilization of MSWI-FA is limited by its fine particle size and the presence of soluble salts and potentially hazardous elements. Consequently, the appropriate replacement level, the method of ash preparation, and its actual chemical composition are of fundamental importance to the properties of the resulting composite.

Compared with MSWI bottom ash and fly ash, considerably fewer studies have addressed boiler dust separated as an individual process stream. Broad reviews of the material utilization of MSWI residues focus mainly on bottom ash, fly ash, and air pollution control residues [3,7,8]. Although boiler dust is distinguished in the European waste classification, including as boiler dust containing dangerous substances (19 01 15*), its use in cementitious materials has been investigated to a much lesser extent. Saikia et al. [24] did investigate a material referred to as boiler and fly ash (BFA) from a fluidized-bed installation; however, before being used as a partial replacement for fine aggregate, it was subjected to chemical treatment aimed, among other purposes, at removing aluminum and sulfate compounds. Individual studies have also indicated the possibility of using separated boiler ash as a partial cement replacement in cement mortars; however, its composition and properties are strongly dependent on the source and configuration of the installation [38]. As demonstrated by Czop et al. [28], who compared materials originating from three different MSWI plants, even residues belonging to the same category may differ in properties relevant to their application in construction materials. The characteristics of the resulting materials are influenced, among other factors, by the morphology and seasonal variability of the incinerated waste, the degree of prior waste segregation, combustion process parameters, grate and boiler design, the collection point of individual fractions, and the flue gas cleaning technology applied. Therefore, a technological solution developed for waste from one installation should not be directly transferred to material originating from another source without renewed characterization and experimental validation.

When considering the use of MSWI process residues in the production of construction materials, it should be emphasized that a substantial proportion of the available studies focus on the utilization of a single type of MSWI residue. A limited number of studies have also investigated the combined use of different MSWI residues as constituents of cementitious materials. Mixtures of bottom ash and APC fly ash have been used as aggregate replacements in non-structural concrete [39], whereas processed combined ash comprising bottom ash and fly ash has been used as a partial or complete replacement for natural aggregate in mortars and concrete [40]. Zhang et al. [34] used a mixture of bottom ash, boiler ash, and air pollution control lime to produce, following thermal treatment at 1100 °C, an eco-admixture intended as a partial cement replacement in concrete. A mixture of MSWI fly ash (19 01 13*) and solid wastes from gas treatment (19 01 07*) has also been investigated as a partial replacement for natural sand in cement mortars [41]. MSWI process residues have additionally been used in combination with waste from the cement industry (CKD or CBPD). Systems have been investigated in which MSWI-FA was used as a sand replacement and cement kiln dust (CKD) as a partial cement replacement [42], as well as systems in which MSWI bottom ash was used as a sand replacement and cement kiln bypass dust (CBPD) as a cement replacement [43]. The results confirmed that mortar properties depend both on the type and content of the waste material and on the proportions of the coexisting constituents. However, these studies do not explain the behavior of different streams generated in a different MSWI technological configuration, particularly when hazardous boiler dust is used. Despite previous attempts to combine several MSWI residues, there remains a lack of systematic studies on systems in which separate streams originating from flue gas treatment and the boiler section are simultaneously used as replacements for two different primary constituents of mortar, namely natural sand and cement.

In the present study, a mixture of different waste streams originating from the Municipal Waste Thermal Treatment Plant in Konin, Poland, was used. The first waste material, designated as MSWI-SW/FA, consisted of a mixture of solid wastes from gas treatment (19 01 07*) and fly ash containing dangerous substances (19 01 13*) and was used as a partial replacement for natural sand. The second waste material, designated as MSWI-BD, was boiler dust containing dangerous substances (19 01 15*) and was used as a partial cement replacement. Both waste materials were incorporated into the cement mortar either separately or simultaneously at different proportions. The aim of this study is to determine the effects of waste type, replacement level, and the relative proportion of MSWI-SW/FA and MSWI-BD on the properties of fresh and hardened cement mortars. The experimental program includes evaluation of fresh mortar consistency, flexural and compressive strength after 7 and 28 days of curing, water absorption, and resistance to cyclic freezing and thawing, supplemented by SEM microstructural analysis for selected mixtures. The novelty of this study does not lie in the general use of MSWI residues in cementitious materials, which has been previously reported, but in the investigation of a plant-specific configuration of hazardous MSWI residue streams originating from a different incineration facility than those considered in our previous studies. In the present study, solid wastes from gas treatment (19 01 07*) and fly ash containing dangerous substances (19 01 13*) form a combined stream, designated as MSWI-SW/FA, while boiler dust containing dangerous substances (19 01 15*) is collected separately as MSWI-BD. These two residue streams are simultaneously used as replacements for two different primary mortar constituents. The systematic variation in their relative proportions further enables the identification of composition-dependent effects that cannot be derived from studies considering individual MSWI residues separately.

## 2. Materials and Methods

### 2.1. Materials

Cement mortars were prepared using CEM II/A-LL 42.5R-NA Portland cement supplied by CEMEX and conforming to PN-EN 197-1 [44]. Standard quartz sand (Kwarcmix) with a particle size range of 0–2 mm, meeting the requirements of PN-EN 196-1, was used as aggregate. Tap water from the municipal water supply network in Czestochowa, complying with PN-EN 1008:2004 [45], was used for mortar preparation.

**Figure 1:**
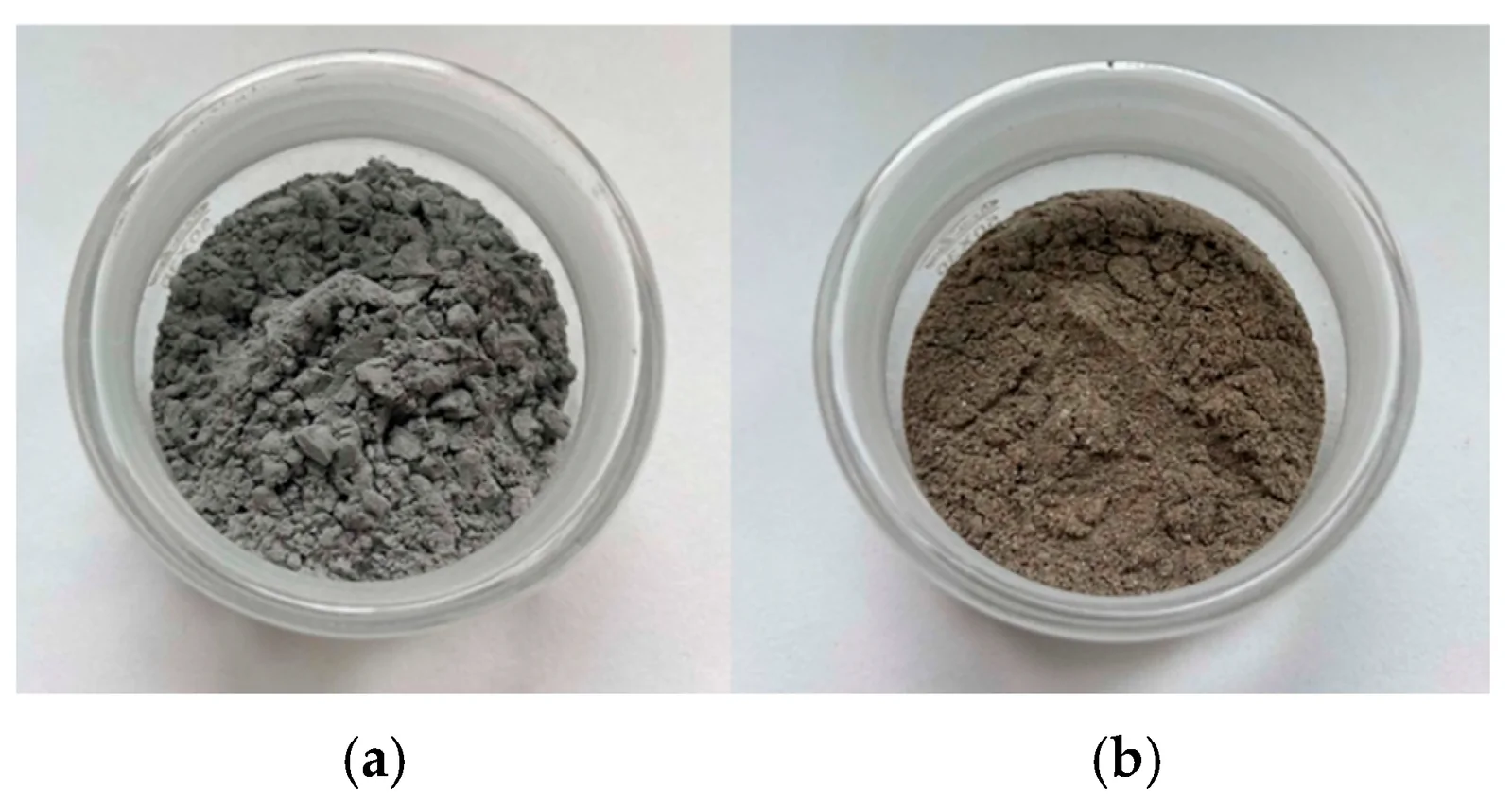
MSWI-SW/FA waste (**a**); MSWI-BD waste (**b**).

In addition, municipal solid waste incineration residues obtained from the Municipal Solid Waste Thermal Treatment Plant in Konin, Poland (ZTUOK), were used as partial substitutes for conventional raw materials. The MSWI-SW/FA was obtained as a combined process stream comprising wastes classified under codes 19 01 07* (solid wastes from gas treatment) and 19 01 13* (fly ash containing hazardous substances) and was used as a partial replacement for natural sand (Figure 1a). The second material, waste classified under code 19 01 15* (boiler dust containing hazardous substances), designated as MSWI-BD, was used as a partial replacement for cement (Figure 1b). The relative proportions of the two waste fractions constituting the MSWI-SW/FA stream were not specified by the plant.

The chemical composition of the waste materials used in the study was determined using an X-ray fluorescence (XRF) spectrometer (Bruker AXS, Karlsruhe, Germany). For Table 1, Ca, Si, S, Al, Mg, Fe, Na, K, and Ti were converted stoichiometrically to their corresponding oxide equivalents. Chlorine and potentially toxic elements were reported as elemental concentrations. The specific densities of the waste materials, determined using the procedure described in PN-EN 196-6 [46], were 2.398 g/cm^3^ for MSWI-BD and 2.432 g/cm^3^ for MSWI-SW/FA, respectively.

**Figure 2:**
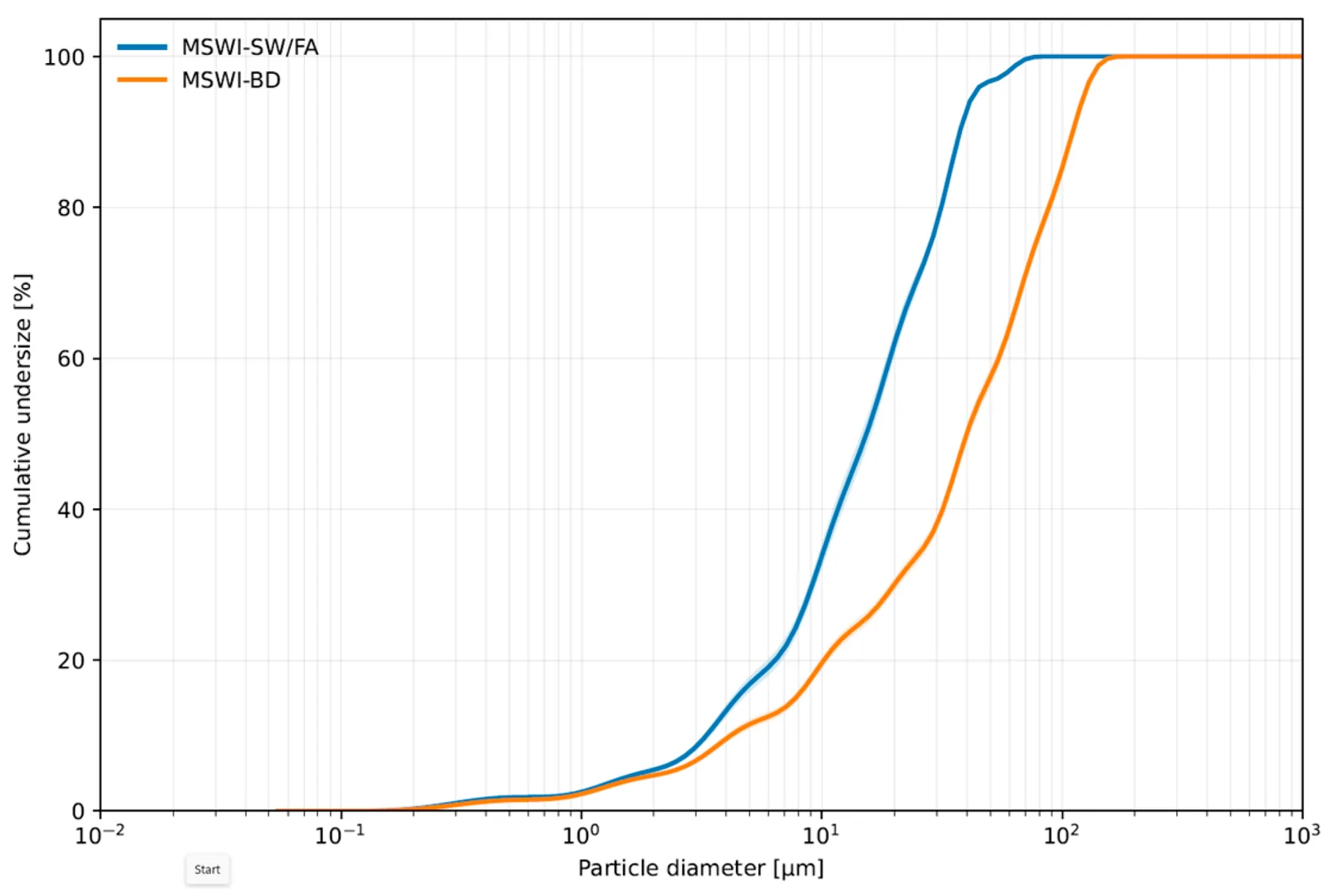
Cumulative particle size distributions of MSWI-SW/FA and MSWI-BD determined by laser diffraction. The curves represent the mean values obtained from three replicate measurements.

The particle size distributions of the MSWI residues were determined by laser diffraction using an ANALYSETTE 22 NeXT Nano (FRITSCH GmbH, Idar-Oberstein, Germany). Measurements were performed in triplicate over a measurement range of 0.01–3800 µm using the Fraunhofer calculation model, with ultrasonic dispersion of the samples prior to measurement. The characteristic particle diameters D10, D50, and D90 were determined from the cumulative particle size distributions. MSWI-SW/FA exhibited D10, D50, and D90 values of 3.45, 15.96, and 39.06 µm, respectively, with 96.44% of the particles below 50 µm and the entire measured fraction below 100 µm (Table 2). In comparison, MSWI-BD showed D10, D50, and D90 values of 4.37, 41.82, and 115.27 µm, respectively, with 56.12% of the particles below 50 µm and 83.49% below 100 µm. MSWI-SW/FA exhibited a considerably finer particle size distribution than MSWI-BD, with D50 values of 15.96 and 41.82 µm and D90 values of 39.06 and 115.27 µm, respectively. Figure 2 shows that the cumulative particle size distribution curve of MSWI-SW/FA is shifted toward smaller particle diameters compared with MSWI-BD, confirming that MSWI-SW/FA is the finer residue.

**Figure 3:**
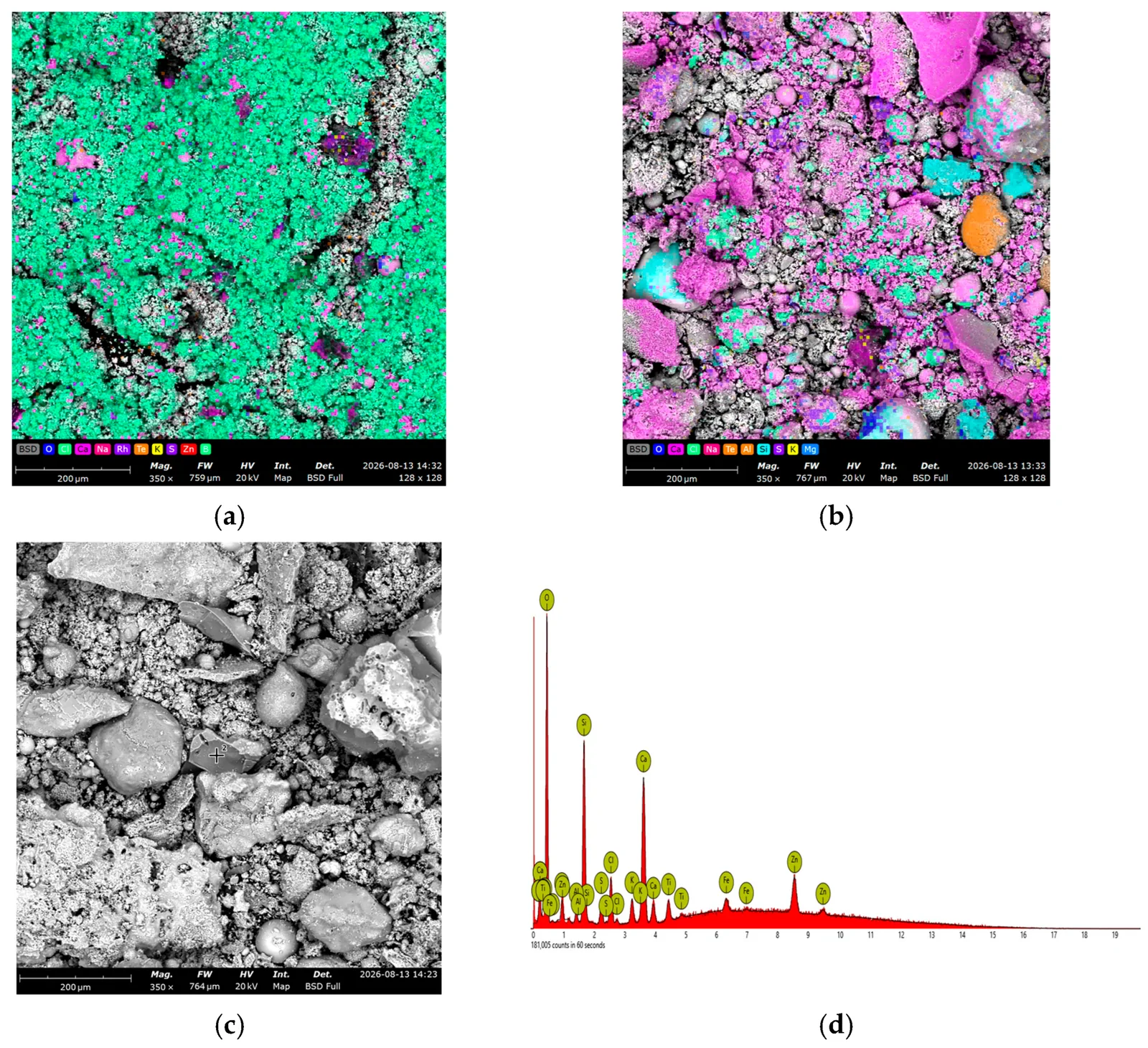
SEM images and EDS analysis of the investigated MSWI residues: (**a**) MSWI-SW/FA, 350×; (**b**) MSWI-BD, 350×; (**c**) MSWI-BD with the location of EDS spot analysis; (**d**) EDS spectrum of spot 2 showing local Zn enrichment.

SEM-EDS observations revealed clear differences in the morphology of the investigated waste materials (Figure 3a,b). SEM observations were performed using a scanning electron microscope (LEO Electron Microscopy Ltd., Cambridge, UK) equipped with an energy-dispersive X-ray spectroscopy (EDS) system (Bruker AXS, Karlsruhe, Germany). MSWI-BD was characterized mainly by smaller, irregular, and partially agglomerated particles, whereas MSWI-SW/FA exhibited a more varied morphology, including agglomerates and larger compact particles. EDS analysis also confirmed differences in the elemental composition of the two materials. MSWI-SW/FA was mainly enriched in Cl, Ca, and Na; in the analyzed area, their contents were approximately 22.9, 15.1, and 9.3 wt.%, respectively (Figure 3a). In the case of MSWI-BD, the EDS spot analyses revealed pronounced local heterogeneity in elemental composition. Therefore, additional point analysis was performed for a selected area (Figure 3c,d). At spot 2, a distinct local enrichment in Zn was observed, with a Zn content of 13.96 wt.%, accompanied by Ca (10.07 wt.%), Si (8.31 wt.%), Cl (2.71 wt.%), and Fe (2.14 wt.%). The spot analyses therefore indicate that MSWI-BD contains particles with markedly different local elemental compositions, confirming its heterogeneous nature at the microscale.

### 2.2. Methods

The experimental program comprised one reference mortar (MS) and 12 mortars modified with MSWI residues (Table 3). The modified mortars were designed to evaluate the individual and combined effects of MSWI-BD and MSWI-SW/FA at nominal dosage levels of 2.93, 10, 17.07, and 20 wt.% relative to the initial cement mass. MSWI-BD was used as a partial cement replacement, with the cement mass reduced by the corresponding amount of MSWI-BD. MSWI-SW/FA was dosed at the same nominal levels relative to the initial cement mass but was used as a volumetric replacement for natural sand. Accordingly, the mass of sand removed from each mixture was adjusted to account for the difference in density between MSWI-SW/FA and the standard quartz sand. The residues were incorporated either individually or simultaneously in different proportions. In the MIX designation, the first value corresponds to the MSWI-BD dosage and the second to the MSWI-SW/FA dosage. For each mortar composition, 21 prismatic specimens measuring 40 × 40 × 160 mm were prepared. The specimens were subjected to a comprehensive evaluation of their mechanical, physical, and performance-related properties. The testing program included the determination of flexural and compressive strength, water absorption, freeze–thaw resistance, and fresh mortar consistency. For the control cement mortars and the mortars containing waste materials, the consistency of the fresh mortars was determined, while the hardened mortars were tested for water absorption, mechanical strength, and freeze–thaw resistance.

The workability of the fresh mortars was evaluated immediately after mixing by determining their consistency using the flow table method in accordance with PN-EN 1015-3 [47]. The flow-table test was performed in triplicate for each mortar composition. The measured values were used to assess the effect of the waste materials on the properties of the fresh mortars, particularly their flowability and behavior during specimen casting.

The water absorption of the mortars was determined after 28 days of curing in accordance with PN-B-04500:1985 [48], using the complete water saturation method. For each mortar mix, three prismatic specimens measuring 40 × 40 × 160 mm were tested.

The mechanical properties of the hardened mortars were determined in accordance with PN-EN 196-1 [49] using an MMC-3742.FPR testing machine (ToniTechnik, Berlin, Germany). The tests were performed after 7 and 28 days of curing. Flexural strength was determined using three prismatic specimens for each mortar mix. The load was increased uniformly at a rate of 50 ± 10 N/s until specimen failure. Subsequently, the resulting prism halves were used to determine compressive strength. The compressive strength test was performed on six half-prisms, with the load increased uniformly at a rate of 2400 ± 200 N/s.

In addition to the absolute compressive strength values, a relative compressive strength index (RSI) was calculated to quantify the mechanical efficiency of MSWI-BD used as a partial cement replacement. The index was determined as the ratio of the compressive strength of the mortar containing MSWI-BD to that of the reference mortar at the same curing age:

$$RSI\;=\;\frac{f_{c,\mathrm{MSWI}-\mathrm{BD}}}{f_{c,\mathrm{ref}}}\;\times \;100\%$$

where f_c,MSWI-BD_ is the compressive strength of the mortar containing MSWI-BD and f_c,ref_ is the compressive strength of the reference mortar.

The index was calculated after 7 and 28 days. It should be emphasized that this parameter is used here as a comparative mechanical indicator and not as a standardized determination of pozzolanic activity.

The resistance of the mortars to cyclic freezing and thawing was evaluated in accordance with the procedure specified in PN-B-04500:1985 [48]. The tests were carried out using a Toropol K-010 climatic chamber (Toropol, Warsaw, Poland). For each mortar mix, 12 prismatic specimens measuring 40 × 40 × 160 mm were prepared. Half of the specimens were subjected to 25 freeze–thaw cycles, while the remaining specimens were retained as reference samples to assess the effect of cyclic freezing and thawing on the mortar properties. Moreover, selected cement mortar specimens were examined using scanning electron microscopy (SEM), enabling analysis of the microstructure of the hardened material and assessment of structural changes resulting from the incorporation of the waste materials.

The results are presented as mean values ± standard deviation (SD), calculated from the replicate measurements performed for each test. Error bars in the figures represent ±1 SD and describe specimen-to-specimen variability. Statistical differences in compressive and flexural strength among mortar series were evaluated using one-way analysis of variance (ANOVA), followed by Tukey’s HSD post-hoc test where appropriate. A significance level of *p* < 0.05 was adopted.

## 3. Results and Discussion

### 3.1. The Consistency of Cement Mortars

**Figure 4:**
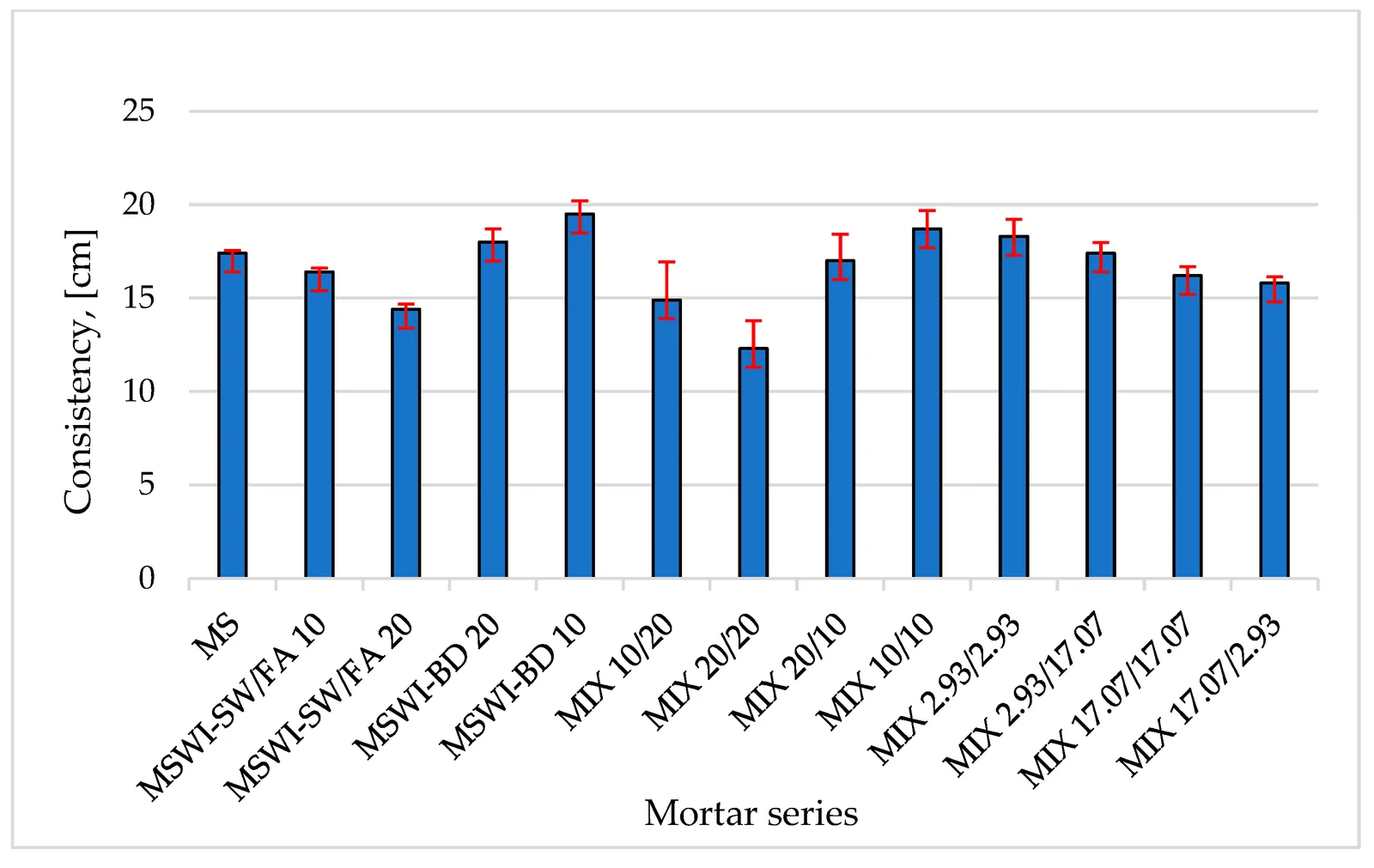
Flow consistency of the individual cement mortar series.

The incorporation of MSWI residues affected the consistency of fresh mortars, with both the direction and magnitude of the changes depending on the type and proportion of the waste material. Replacing sand with MSWI-SW/FA reduced the flow from 17.4 cm for the reference mortar to 16.4 and 14.4 cm at substitution levels of 10 and 20%, respectively. For MSWI-BD, the corresponding flow values were 19.5 and 18.0 cm at 10 and 20% substitution, respectively. Mortars containing both waste materials exhibited flow values ranging from 12.3 to 18.7 cm, indicating that consistency was governed not only by the total waste content but also by the relative proportions of the two residues (Figure 4). The greatest reduction in flow was observed for MIX 20/20, whereas at low substitution levels, the consistency remained close to that of the reference mortar.

The observed decrease in flow with increasing MSWI-SW/FA content is consistent with our previous studies [42] on MSWI residues used as partial sand replacements. In particular, mortars containing 10–20% MSWI-FA (19 01 13*) exhibited a progressive reduction in flow, with values of approximately 14.6–14.9 cm reported at comparable substitution levels. However, the comparison should be treated qualitatively, since the MSWI fly ash investigated in our previous study originated from a different incineration plant and was collected as a separate process stream, whereas in the present study it forms part of the combined MSWI-SW/FA stream together with solid wastes from gas treatment (19 01 07*). The reduction in flow observed with increasing MSWI-SW/FA content can be related to its relatively small particle size, with D50 = 15.96 µm and D90 = 39.06 µm, together with the irregular and agglomerated morphology observed by SEM. The high proportion of particles below 50 µm (96.44%) may contribute to increased water demand and reduced mortar flowability.

### 3.2. Mechanical Properties of Cement Mortars

**Figure 5:**
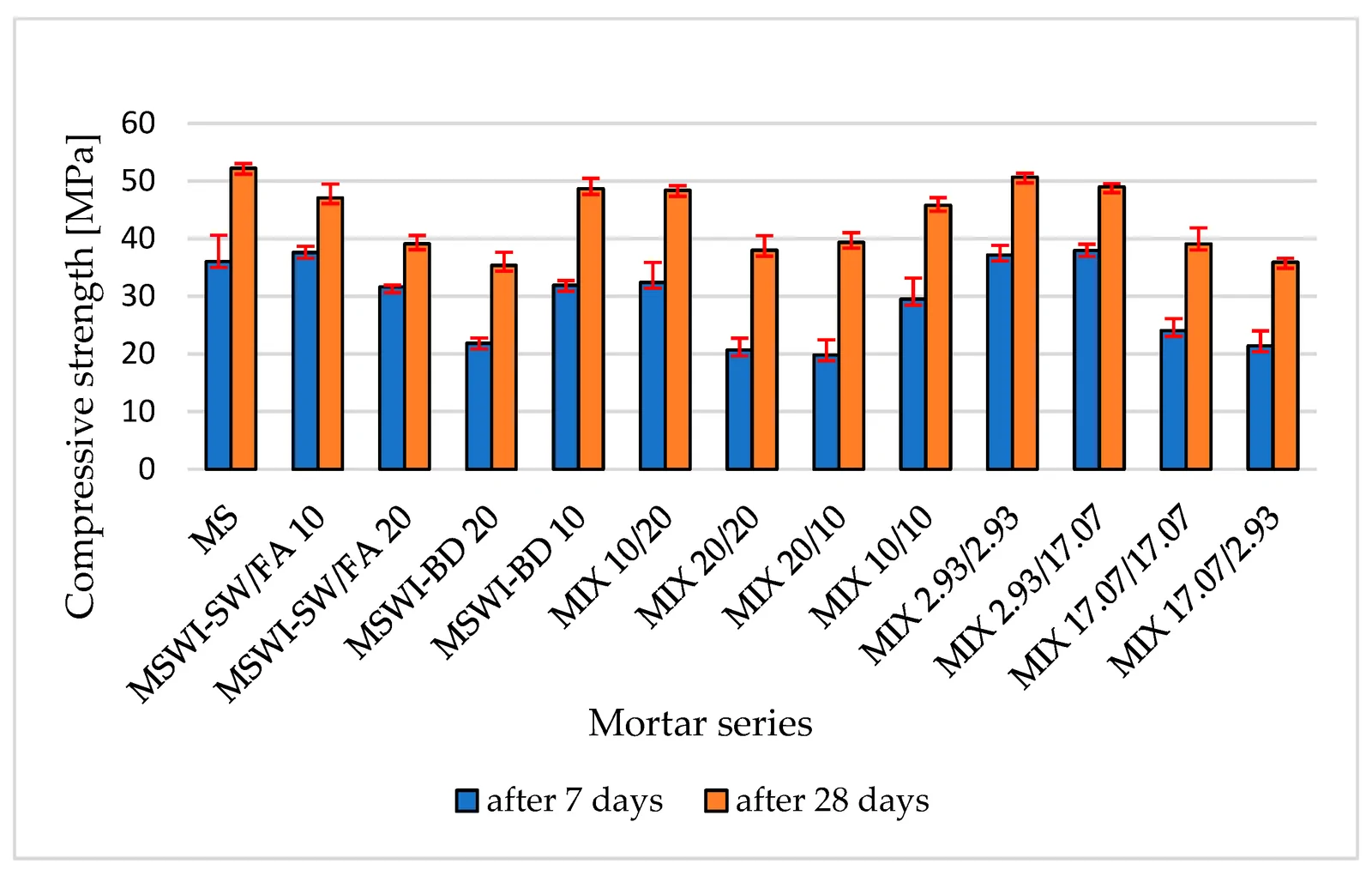
Compressive strength of the individual cement mortar series after 7 and 28 days of curing.

The mechanical properties of the hardened cement mortars were determined. Compressive strength was strongly dependent on the type and level of substitution (Figure 5). After 28 days, the reference mortar reached a compressive strength of 52.23 MPa. For mortars containing a single waste material, increasing the MSWI-SW/FA content from 10 to 20% reduced the compressive strength from 47.08 to 39.12 MPa, whereas the corresponding values for MSWI-BD were 48.69 and 35.37 MPa. These results indicate that high substitution levels are detrimental to compressive strength, with the effect being particularly pronounced when cement is replaced with MSWI-BD. The stronger reduction in mechanical strength caused by MSWI-BD can primarily be attributed to its role as a cement replacement. Increasing the MSWI-BD content progressively decreases the amount of hydraulic binder available for the formation of the load-bearing cementitious matrix. In contrast, MSWI-SW/FA replaces natural sand while the cement content remains unchanged, which may explain its less pronounced effect on mechanical strength. The finer particle size distribution of MSWI-SW/FA may additionally influence particle packing and the fresh-state behavior of the mortar; however, the present results do not allow this effect to be separated from the influence of residue composition and replacement mode.

To further assess the mechanical contribution of MSWI-BD, the relative compressive strength index (RSI) was calculated with respect to the reference mortar. After 28 days, the index reached 93.2% for MSWI-BD 10 and 67.7% for MSWI-BD 20. Thus, at a 10% cement replacement level, the mortar retained more than 90% of the reference compressive strength, whereas increasing the MSWI-BD content to 20% resulted in a substantially lower relative strength. However, the relative strength index should not be interpreted as direct evidence of pozzolanic or hydraulic reactivity of MSWI-BD, since the observed mechanical response may result from the combined effects of cement dilution, physical filling and particle packing, nucleation effects, and possible chemical reactions. The present experimental program does not allow these mechanisms to be quantitatively distinguished. Therefore, the relatively high strength retained at the 10% MSWI-BD replacement level should be interpreted only as evidence of favorable mechanical performance, rather than as confirmation of pozzolanic activity.

The most favorable results were obtained for mixtures containing a low proportion of MSWI-BD. After 28 days, MIX 2.93/2.93 reached 50.68 MPa, corresponding to 97.0% of the reference mortar strength, while MIX 2.93/17.07 reached 49.01 MPa, equivalent to 93.8% of the MS value. The results therefore indicate that the effect of the two waste materials is not a simple function of their total content, but depends on their relative proportions. In particular, limiting the MSWI-BD content allows a higher proportion of MSWI-SW/FA to be incorporated without a substantial reduction in compressive strength.

**Figure 6:**
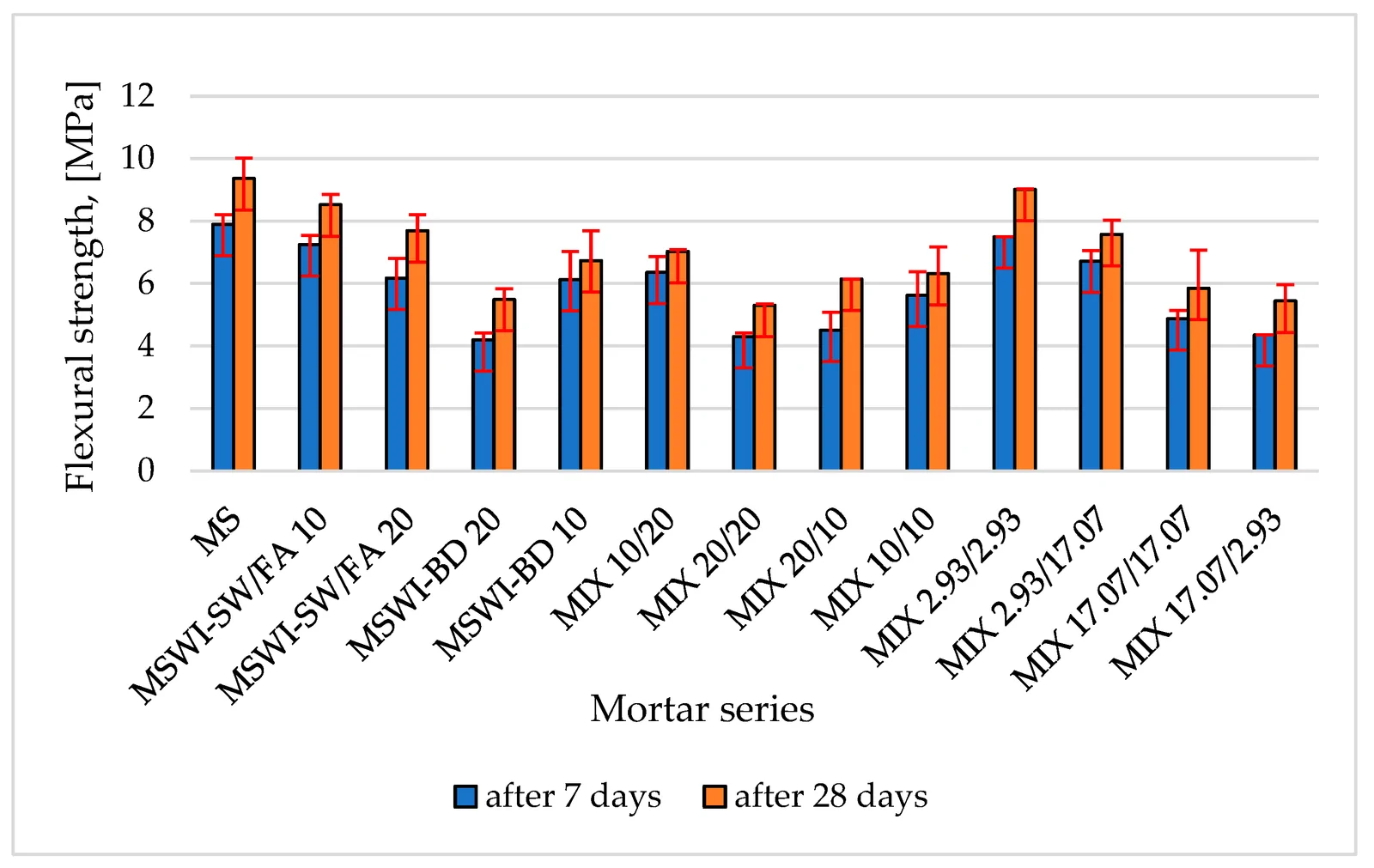
Flexural strength of the individual cement mortar series after 7 and 28 days of curing.

A similar trend was observed for flexural strength (Figure 6), although the effect of MSWI-BD was more pronounced in this case. After 28 days, the reference mortar reached 9.36 MPa, whereas the incorporation of 10 and 20% MSWI-BD reduced the flexural strength to 6.73 and 5.49 MPa, respectively. For MSWI-SW/FA, the decrease was less pronounced, with corresponding values of 8.52 and 7.69 MPa. The most favorable variant containing both waste materials was MIX 2.93/2.93, which reached 9.01 MPa, corresponding to 96.3% of the reference mortar value. These results confirm that a low substitution level allows mechanical properties close to those of the reference mortar to be maintained, whereas increasing the MSWI-BD content leads to a marked deterioration in flexural strength. Statistical analysis confirmed a significant effect of mortar composition on both compressive and flexural strength at 7 and 28 days (ANOVA, *p* < 0.001). Tukey’s HSD test showed that selected mixtures with low MSWI-BD content did not differ significantly from the reference mortar, whereas mixtures containing higher proportions of MSWI-BD generally exhibited significantly lower mechanical strength.

The observed decrease in strength with increasing MSWI-BD content is consistent with the general trends reported for MSWI residues used as partial cement replacements. Zheng et al. [33] demonstrated that increasing the content of MSWI fly ash used as a cement replacement led to a deterioration in mechanical properties, whereas using the waste as a sand replacement had a less pronounced effect on strength. Similarly, Tokgoz et al. [38] reported a reduction in mortar strength with increasing contents of untreated boiler ash used as a cement replacement. However, these comparisons should be treated qualitatively, since the composition and properties of MSWI residues strongly depend on their source and incineration technology.

Previous studies by the authors [41,42,43], conducted using different MSWI residue streams originating from other incineration systems, also showed that the effect of waste incorporation on mortar strength depends strongly on the type of residue, its function in the mixture, and the replacement level. Therefore, direct quantitative comparison with the present results is not appropriate. Nevertheless, these studies support the general observation that the performance of waste-modified mortars is strongly dependent on the material type and source of origin.

To place the obtained results in a broader context, Table 4 compares the 28-day compressive strength of cement mortars incorporating different waste-derived materials reported in the literature. The comparison includes recycled ceramic aggregate [50], waste glass [51], waste marble powder [52], and selected municipal solid waste incineration (MSWI) residues [38,41]. Relative compressive strength was calculated as the ratio of the 28-day compressive strength of the modified mortar to that of the corresponding reference mortar, multiplied by 100%.

The results indicate that compressive strength retention depends on the type of waste material, its role in the mixture, and the replacement level. In the present study, MIX 2.93/2.93 achieved a compressive strength of 50.68 MPa, corresponding to 97.0% of the reference mortar strength (52.23 MPa). However, differences in mixture composition, binder type, replacement basis (mass or volume), and testing procedures limit direct quantitative comparisons between studies.

### 3.3. The Freeze-Thaw Resistance of Cement Mortars

**Figure 7:**
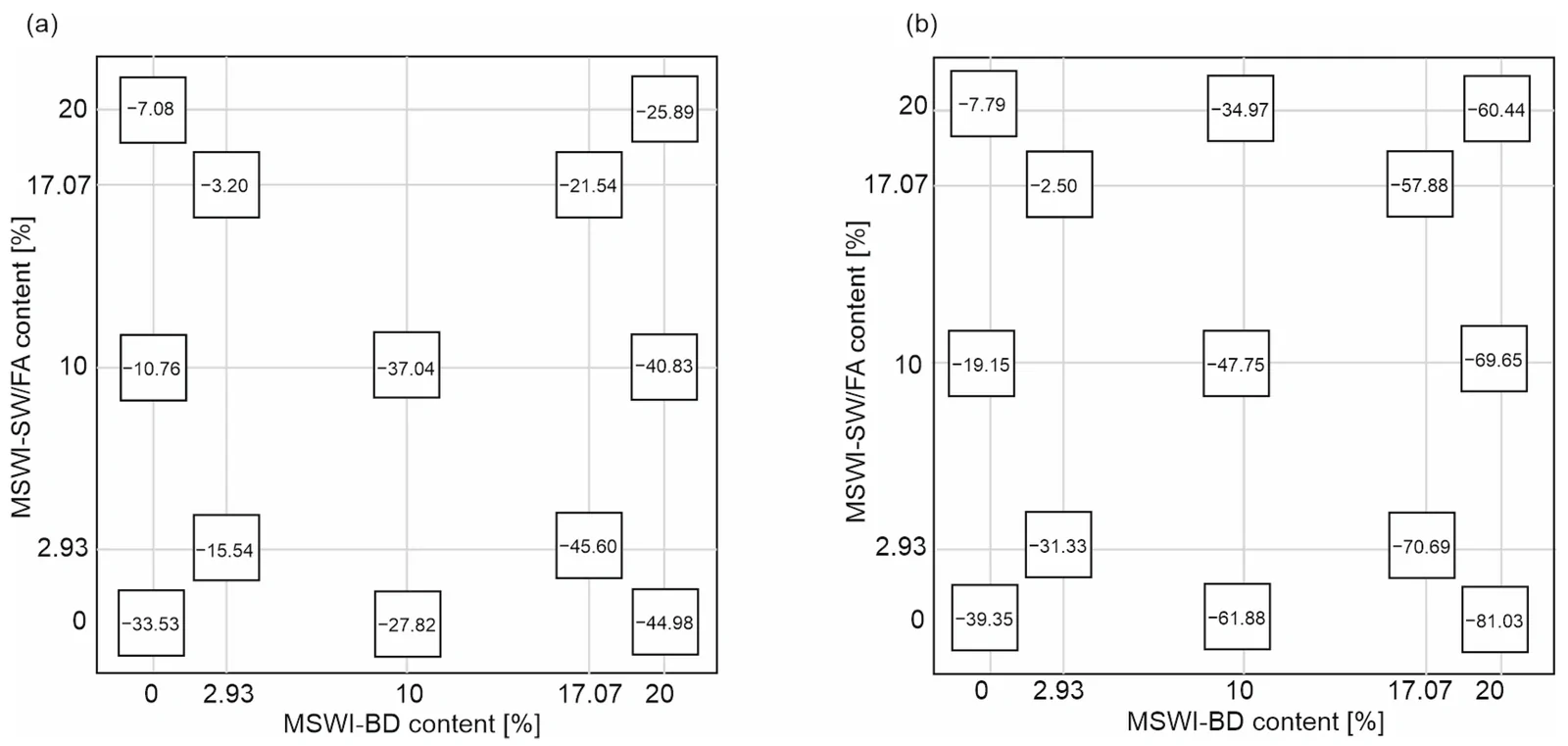
Effect of MSWI-BD and MSWI-SW/FA contents on the percentage changes in mechanical strength of cement mortars after 25 freeze–thaw cycles: (**a**) compressive strength; (**b**) flexural strength.

The percentage changes in compressive and flexural strength after 25 freeze–thaw cycles are presented in Figure 7a,b. A clear composition-dependent response was observed. The reference mortar exhibited reductions of 33.53% in compressive strength and 39.35% in flexural strength. Mortars containing MSWI-SW/FA showed considerably lower losses, decreasing to 7.08% and 7.79%, respectively, at 20% substitution. In contrast, increasing the MSWI-BD content adversely affected freeze–thaw resistance; at 20% substitution, the losses reached 44.98% in compressive strength and 81.03% in flexural strength. Overall, the results indicate that freeze–thaw resistance is governed not by the total MSWI residue content alone, but primarily by the type and relative proportion of the incorporated residues. Among the mixtures containing both residues, the relative proportions were decisive. MIX 2.93/17.07 exhibited the most favorable behavior, with only a 3.20% reduction in compressive strength and a 2.5% reduction in flexural strength after freeze–thaw exposure. Conversely, mixtures with a high MSWI-BD content showed markedly greater deterioration. The deterioration in freeze–thaw resistance at high MSWI-BD contents is consistent with the increased water absorption of these mixtures. A larger amount of water accessible within the hardened matrix may generate higher internal stresses upon freezing and thereby promote progressive damage during cyclic exposure. In contrast, mixtures containing a low MSWI-BD content and a higher proportion of MSWI-SW/FA maintained relatively low water absorption and exhibited substantially smaller strength losses after freeze–thaw cycling. These results indicate that freeze–thaw resistance depends not only on the total MSWI residue content but primarily on the type and relative proportion of the incorporated residues. Similar variability in the freeze–thaw resistance of cementitious materials containing MSWI residues has been reported in previous studies. Thomas and Slosarczyk [29] showed that the use of MSWI slag as an aggregate replacement did not necessarily deteriorate freeze–thaw resistance, although microcracking and local spalling were observed after cyclic exposure. Vilarinho et al. [25] also reported favorable freeze–thaw performance for selected mortars containing MSWI bottom ash. These findings support the present results, indicating that freeze–thaw resistance depends strongly on the type, content, and function of the incorporated MSWI residue.

### 3.4. The Water Absorption of Cement Mortars

**Figure 8:**
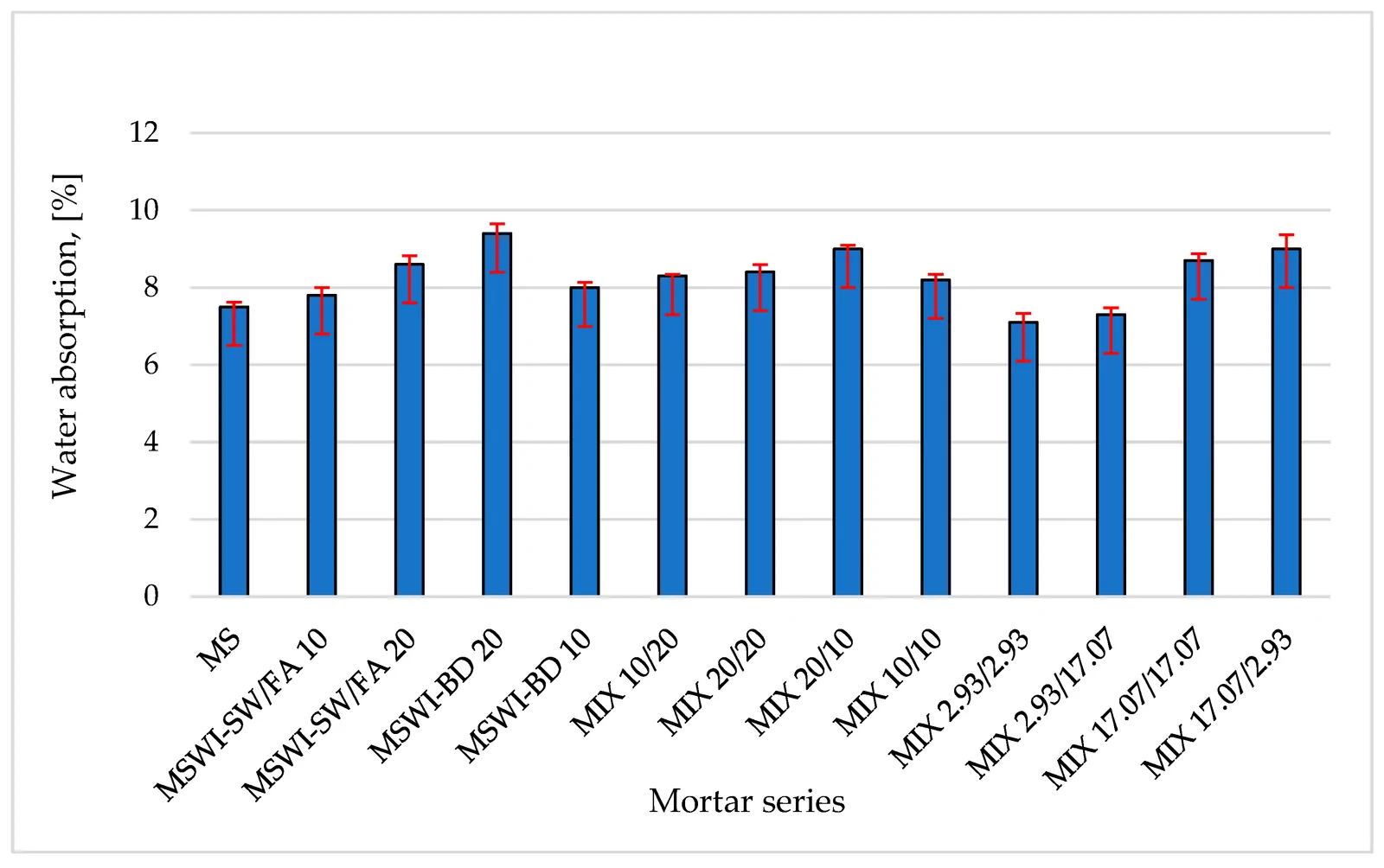
Water absorption of the individual cement mortar series after 28 days of curing.

Water absorption of the mortars depended primarily on the substitution level and the proportion of MSWI-BD (Figure 8). The reference mortar exhibited a water absorption of 7.5%, whereas increasing the MSWI-BD content from 10 to 20% resulted in an increase from 8.0 to 9.4%. Among the mixtures containing both waste materials, the lowest value was obtained for MIX 2.93/2.93 (7.1%), while MIX 2.93/17.07 exhibited a water absorption of 7.3%, remaining close to that of the reference mortar. In contrast, mixtures with high MSWI-BD contents reached values of approximately 9%. The increase in water absorption with increasing MSWI-BD content is consistent with the progressive reduction in cement content and indicates the formation of a matrix containing a greater proportion of water-accessible voids. This interpretation is also consistent with the simultaneous decrease in mechanical strength observed for these mixtures. Although MSWI-BD had a coarser particle size distribution than MSWI-SW/FA, the present results suggest that the increase in water absorption was governed primarily by the reduced cement content and resulting matrix structure rather than by particle size alone.

### 3.5. Microstructural Analysis of Selected Mortars

**Figure 9:**
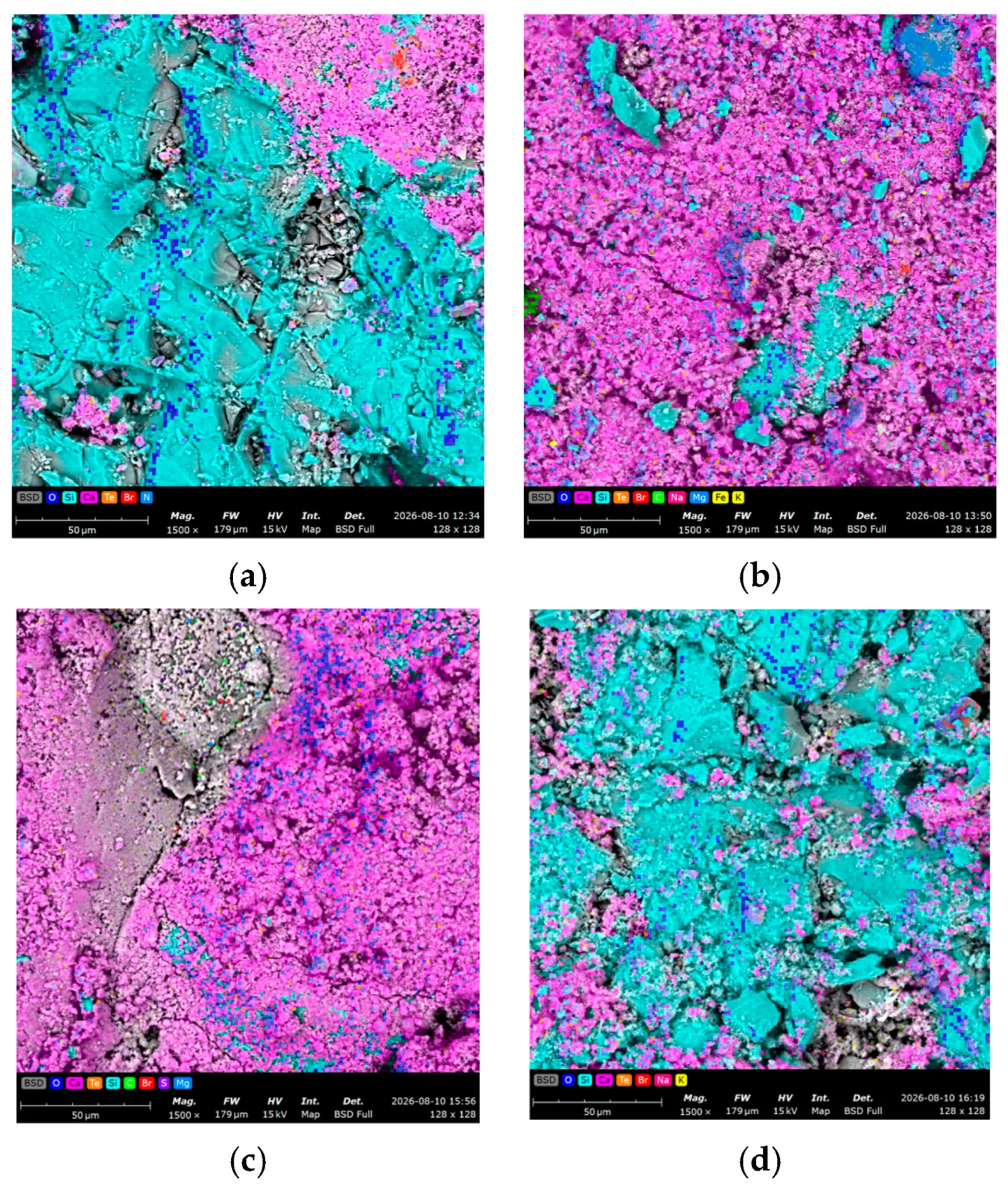
SEM-EDS combined elemental maps (1500×) of selected cement mortars: (**a**) MS; (**b**) MSWI-BD 10; (**c**) MIX 2.93/2.93; (**d**) MIX 2.93/17.07.

Representative SEM-EDS combined elemental maps of the reference mortar and selected waste-modified mortars are presented in Figure 9. SEM-EDS observations revealed distinct microstructural and compositional differences between the investigated mortar series. The reference mortar was characterized by regions dominated by Si-rich angular particles associated primarily with quartz aggregate. The incorporation of MSWI residues increased the chemical heterogeneity of the matrix. In mortars containing a higher proportion of MSWI-BD, the analyzed regions showed a Ca-rich matrix with a relatively low local Si contribution, whereas mortars containing a greater proportion of MSWI-SW/FA exhibited more pronounced Si-rich regions and irregular siliceous particles, most likely associated predominantly with residual quartz aggregate. Particularly noteworthy was the mortar containing low proportions of both residues (MIX 2.93/2.93), in which the examined region was dominated by a relatively homogeneous Ca-rich matrix. This observation is consistent with the comparatively low water absorption and mechanical properties close to those of the reference mortar. In contrast, increasing the contribution of MSWI-SW/FA while maintaining a low MSWI-BD content resulted in a more Si-rich and heterogeneous microstructure. The SEM-EDS observations are consistent with the macroscopic performance of the selected mortars. In particular, MIX 2.93/2.93 exhibited a comparatively uniform Ca-rich matrix together with mechanical properties and water absorption values close to those of the reference mortar, whereas MIX 2.93/17.07 showed greater local chemical heterogeneity. These observations indicate that the microstructure of the mortars depends not only on the total amount of MSWI residues but also on the type and relative proportion of the incorporated waste streams. However, the present SEM–EDS analysis provides only qualitative support for these relationships and does not allow direct identification of changes in hydration products or quantitative characterization of the pore structure.

The observed microstructural differences are generally consistent with previous studies on cement mortars containing MSWI residues. Poranek et al. [32], based on SEM–EDS observations of mortars containing MSWI fly ash, reported that at relatively low waste contents the overall microstructure of the modified mortar remained comparable to that of the reference material. A similar tendency can be observed in the present study for MIX 2.93/2.93, which exhibited a relatively homogeneous Ca-rich matrix and mechanical properties close to those of the reference mortar. With increasing MSWI residue content, however, the local chemical heterogeneity became more pronounced, as particularly evident for MIX 2.93/17.07, where Si-rich regions were observed. These comparisons should remain qualitative because the chemical composition, treatment, and mode of incorporation of MSWI residues differ between studies. This interpretation is also consistent with Zheng et al. [33], who demonstrated that the effect of MSWI fly ash on mortar performance depends strongly on whether the residue is used as a cement or sand replacement, with cement substitution having a more detrimental effect on mechanical performance.

### 3.6. Environmental Safety and Leaching Considerations

The environmental safety of cementitious materials containing MSWI residues is closely related to the potential release of chlorides, sulfates, and potentially toxic elements. Previous studies have shown that cement-based matrices can significantly reduce the mobility of hazardous constituents originating from MSWI residues. For example, immobilization levels exceeding 98% for chlorides and approximately 96% for sulfates have been reported in cement-based matrices containing MSWI ashes [9]. High immobilization of potentially toxic metal ions has also been reported in related MSWI-containing cementitious systems [53,54]. In our previous study, leaching tests of hardened cement mortars containing MSWI-FA and MSWI-SW showed low concentrations of most investigated metal ions; for the mortar containing both residues, Zn and Pb concentrations were 0.112 and 0.221 mg/dm^3^, respectively, while Cu was below 0.01 mg/dm^3^. However, Ba reached 3.682 mg/dm^3^ [41]. In the present study, the Ba content of the raw residues was relatively low, amounting to 0.115 wt.% in MSWI-BD and 0.027 wt.% in MSWI-SW/FA. This difference further illustrates the source-dependent character of MSWI residues and supports the need to avoid direct extrapolation of leaching behavior between materials originating from different incineration facilities.

Elevated Ba leachability has also been reported by other authors, with values as high as 596 mg/dm^3^ for MSWI-FA-containing systems [32]. These findings confirm that the environmental behavior of MSWI residues is strongly dependent on their source and chemical composition. Therefore, results obtained for materials from other incineration facilities cannot be directly extrapolated to the residues investigated here. The present study did not include dedicated leaching tests; therefore, further work should address the environmental performance of the developed mortars, particularly with respect to the release of soluble salts and potentially toxic elements.

## 4. Conclusions

This study demonstrated that the properties of cement mortars containing MSWI residues depend strongly on the type, substitution level, and relative proportion of the incorporated waste streams. The main conclusions are as follows:

(1) Increasing the MSWI-BD content adversely affected mortar performance, resulting in lower mechanical strength and higher water absorption. In contrast, MSWI-SW/FA had a less detrimental effect on strength and generally improved freeze-thaw resistance.
(2) Low substitution levels provided the most favorable mechanical performance. MIX 2.93/2.93 reached 50.68 MPa in compressive strength and 9.01 MPa in flexural strength after 28 days, corresponding to approximately 97% and 96% of the reference values, respectively.
(3) The relative proportion of the residues was more important than their total content. MIX 2.93/17.07 maintained a high compressive strength (49.01 MPa) while exhibiting the best freeze–thaw performance, with only a 3.20% reduction in compressive strength after 25 cycles compared with 33.53% for the reference mortar.
(4) SEM observations revealed composition-dependent microstructural differences among the selected mortars, which were consistent with the observed differences in their mechanical and durability-related properties.

The partial replacement of cement and natural sand with appropriately selected MSWI residues may reduce the consumption of primary raw materials while supporting their valorization as secondary resources in cement-based materials. Due to the source-dependent variability of MSWI residues, upscaling should include source-specific characterization and quality control of the residues before defining suitable replacement levels. However, implementation requires further verification of long-term durability and environmental safety, particularly with regard to the leaching of potentially hazardous constituents.

## Figures and Tables

**Table 1:** Chemical composition of the investigated MSWI residues [wt.%]. Major constituents are expressed as oxide equivalents, whereas Cl and potentially toxic elements are reported as elemental concentrations.

Chemical Composition, [%]	Waste	
	MSWI-SW/FA	MSWI-BD
CaO	34.86	28.36
SiO_2_	3.59	18.92
SO_3_	7.79	14.10
Al_2_O_3_	<0.01	6.17
MgO	2.89	4.08
Fe_2_O_3_	0.73	2.52
Na_2_O	9.57	<0.18
K_2_O	4.77	2.66
TiO_2_	0.31	1.89
Cl	28.81	6.33
Zn	1.71	0.926
Ba	0.027	0.115
Sb	0.088	0.042
Pb	0.200	0.053
Cu	0.068	0.040
Ni	0.002	0.006
Cd	0.015	0.004
Cr	0.011	0.048

**Table 2:** Particle size distribution parameters of the investigated MSWI residues.

Waste Material	D10 [µm]	D50 [µm]	D90 [µm]	<10 µm [%]	<50 µm [%]	<100 µm [%]
MSWI-SW/FA	3.45	15.96	39.06	32.05	96.44	100
MSWI-BD	4.37	41.82	115.27	18.78	56.12	83.49

**Table 3:** The composition of the designed mortars.

Mortar Series	Ingredients				
	Cement	Sand	Water	MSWI-BD	MSWI-SW/FA
	[g]	[g]	[cm^3^]	[g]	[g]
MS	450	1350.0	225	-	-
MSWI-SW/FA 10	450	1300.97	225	-	45.0
MSWI-SW/FA 20	450	1251.93	225	-	90.0
MSWI-BD 20	360	1350.0	225	90.0	-
MSWI-BD 10	405	1350.0	225	45.0	-
MIX 10/20	405	1251.93	225	45.0	90.0
MIX 20/20	360	1251.93	225	90.0	90.0
MIX 20/10	360	1300.97	225	90.0	45.0
MIX 10/10	405	1300.97	225	45.0	45.0
MIX 2.93/2.93	436.815	1335.63	225	13.185	13.185
MIX 2.93/17.07	436.815	1266.30	225	13.185	76.815
MIX 17.07/17.07	373.185	1266.30	225	76.815	76.815
MIX 17.07/2.93	373.185	1335.63	225	76.815	13.185
MIX = MSWI-BD/MSWI-SW/FA					

**Table 4:** Comparison of the 28-day compressive strength of cement mortars incorporating different waste-derived materials.

Waste Material	Replacement of	Replacement Level	28-Day Compressive Strength: Modified/Reference (MPa)	Relative Strength (%)	Ref.
Recycled ceramic aggregate	Natural sand	20 vol.%	42.23/50.21	84.1	[50]
Mixed municipal waste glass	Natural sand	20%	47.43/52.72	90.0	[51]
Waste marble powder	Cement	10 wt.%	52.70/58.60	89.9	[52]
MSWI boiler ash	Cement	20 wt.%	13.00/15.00	86.7	[38]
MSWI fly ash and flue gas treatment residues	Natural sand	20% + 20%	49.78/50.42	98.7	[41]
MSWI-SW/FA and MSWI-BD	Sand and cement	2.93% + 2.93%	42.23/50.21	97.0	Present study

## Data Availability

The data presented in this study are available on request from the corresponding author.
